# The Role of the IL-23/IL-17 Axis in Disease Initiation in Spondyloarthritis: Lessons Learned From Animal Models

**DOI:** 10.3389/fimmu.2021.618581

**Published:** 2021-06-29

**Authors:** Mohamed Mandour, Sijia Chen, Marleen G. H. van de Sande

**Affiliations:** ^1^ Department of Clinical Immunology and Rheumatology, Amsterdam Rheumatology & Immunology Center (ARC), Amsterdam University Medical Centers, Location Academic Medical Center, University of Amsterdam, Amsterdam, Netherlands; ^2^ Department of Experimental Immunology, Infection and Immunity Institute, Amsterdam University Medical Centers, Location AMC, University of Amsterdam, Amsterdam, Netherlands; ^3^ Division of Rheumatology, Inflammation, and Immunity, Brigham and Women’s Hospital and Harvard Medical School, Boston, MA, United States

**Keywords:** spondyloarthritis, interleukin-23/IL-17 axis, HLA-B27, animal models, psoriatic arthritis

## Abstract

Spondyloarthritis (SpA) is a spectrum of chronic inflammatory joint diseases that frequently presents with inflammation of the axial skeleton, peripheral joints, entheses, skin, and gut. Understanding SpA pathogenesis has been proven challenging due to the limited availability of human target tissues. In recent years, the interleukin (IL)-23/IL-17 pathway has been implicated in the pathogenesis of SpA, in addition to the Tumor Necrosis Factor Alpha (TNF-α) cytokine. The underlying molecular mechanisms by which the IL-23/IL-17 pathway triggers disease initiation, both in the joints as well as at extra-musculoskeletal sites, are not precisely known. Animal models that resemble pathological features of human SpA have provided possibilities for in-depth molecular analyses of target tissues during various phases of the disease, including the pre-clinical initiation phase of the disease before arthritis and spondylitis are clinically present. Herein, we summarize recent insights gained in SpA animal models on the role of the IL-23/IL-17 pathway in immune activation across affected sites in SpA, which include the joint, entheses, gut and skin. We discuss how local activation of the IL-23/IL-17 axis may contribute to the development of tissue inflammation and the onset of clinically manifest SpA. The overall aim is to provide the reader with an overview of how the IL-23/IL-17 axis could contribute to the onset of SpA pathogenesis. We discuss how insights from animal studies into the initiation phase of disease could instruct validation studies in at-risk individuals and thereby provide a perspective for potential future preventive treatment.

## Introduction

**Figure 1:**
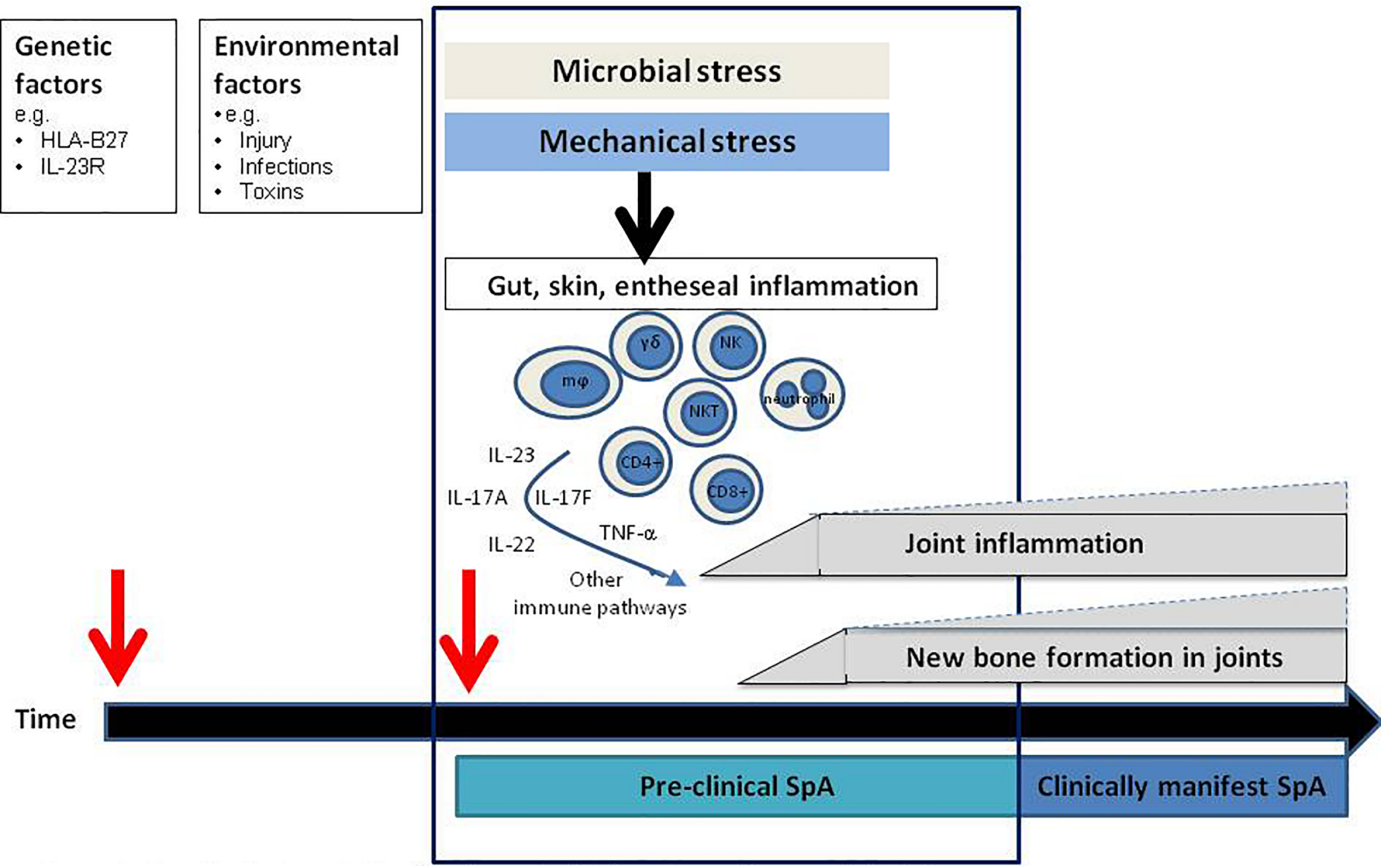
Hypothetical model for the disease initiation phase: the pre-clinical phase. In the pre-clinical phase of disease innate immune triggering results in activation of specific pathogenic immune pathways site distant from the joints resulting in (subclinical) joint inflammation and new bone formation which subsequently develops into clinically manifest spondyloarthritis (SpA). mϕ, macrophages, NK, NK cells; NKT, NKT cells; γδ, gamma delta T cells.

Spondyloarthritis (SpA) is a chronic inflammatory joint disease characterized by inflammation and new bone formation which results in structural damage. Clinically, patients present with axial manifestations, such as sacroiliitis or spondylitis, and/or peripheral manifestations such as arthritis, dactylitis, or enthesitis. Besides, extra-musculoskeletal features, including psoriasis, inflammatory bowel disease, and uveitis, can be present (1). SpA comprises 2 subtypes: peripheral SpA, with psoriatic arthritis (PsA) as the prototypical disease, and axial SpA, which encompasses radiographic axSpA, in which patients have signs of sacroiliitis on X-ray and fulfill the modified New York criteria (2), and the non-radiographic type (3). The disease pathogenesis of SpA is incompletely understood. Genetic factors combined with exposure to microbes by the loss of barrier function in the skin or the gut, or local mechanical stress at entheseal sites, are suggested to induce an inflammatory cascade resulting in joint inflammation and new bone formation. HLA-B27 is the strongest genetic factor linked to SpA. Genetic association studies, animal studies, human ex-vivo and intervention studies have demonstrated that the tumor necrosis factor alpha (TNF-α) and interleukin (IL)-23/IL-17 pathways are key players in the inflammatory cascade, both in the initiation phase of SpA, and during chronic persistent disease (4–6) (**Figure 1**).

TNF-α is a pleiotropic, pro-inflammatory cytokine playing major roles in protection against infections and driving inflammation in immune mediated inflammatory diseases including SpA. It is produced by various immune (activated T cells, macrophages, monocytes, neutrophils) and non-immune cells (fibroblasts, endothelial cells) (7). TNF-α is produced as a transmembrane bound protein expressed on the cell surface of various cell types including lymphocytes and macrophages, which is cleaved into a soluble form by metalloprotease TNF-α converting enzyme (TACE), also called A Disintegrin and Metalloprotease 17 (ADAM17). Both the transmembrane and soluble forms of TNF-α are biologically active and can bind to the TNF-α receptors 1 and 2, but their downstream effects vary (8, 9).

IL-17A is a pro-inflammatory cytokine implicated in various inflammatory disorders. IL-17A by itself only has a modest proinflammatory effect but acts as a potent enhancer of inflammation through synergy with other proinflammatory cytokines such as TNF-α (10). IL-17A was the first to be characterized among the 6 conserved IL-17 proteins (IL-17A-F), followed by IL-17F, which is for 50% structurally similar to IL-17A (11, 12). The IL-17 receptor family comprises the subunits IL-17RA, IL-17RB, IL-17RC, IL-17RD, and IL-17RE. After binding of IL-17A or F to their receptors, Act1 associates with the IL-17 receptor resulting in the activation of various downstream signaling pathways (13). IL-23 is the canonical cytokine that activates IL-17A production. It is a heterodimeric cytokine, which contains a P19 and a P40 subunit. The P19 unit is exclusive to IL-23 whereas the P40 subunit is shared with IL-12 (14). IL-23 signals through the IL-23R and IL-12β1 subunits resulting in activation of the JAK-STAT pathway mainly *via* STAT3 (15). More recently, it has been shown that T cells and innate (-like) lymphocytes can produce IL-17A in response to cytokines other than canonical IL-23. These alternative cytokines include IL-7 and IL-9, which classically are required to maintain peripheral innate(-like) T cell populations (16–18). Other diverse innate immune cells also produce IL-17, including γδ T cells, NKp46+ NK cells, intestinal Paneth cells, invariant natural killer T cells (iNKT), MAIT cells and neutrophils (19). The emergence of distinct pathways culminating in the secretion of IL-17A, in addition to the canonical IL-23/IL-17 pathway, underscores the importance of IL-17A in human health and disease (20). In SpA, IL-17A driven inflammation contributes to erosive joint damage and pathological new bone formation (21).

Although the IL-23/IL-17 axis has a central role in SpA pathology, antibodies targeting IL-17A and IL-23 have demonstrated different levels of efficacy in the various subtypes of SpA (22–24). Most strikingly, IL-23 inhibition in human radiographic axSpA patients was not effective (25), whereas IL-17A-blocking therapies decreased inflammation and disease severity (6, 26). These clinical findings are supported by findings in the *Mycobacterium* tuberculosis (*M.tb*) accelerated HLA-B27 transgenic rat (27) model. The disease phenotype in this model resembles human SpA as these rats develop signs of arthritis and spondylitis, with inflammation and new bone formation. When IL-23 inhibition was started after the rats had developed established disease with arthritis and spondylitis, there was a lack of efficacy just as observed in patients (25, 28). In contrast, blocking IL-23R just after immunizing the rats completely prevented inflammation and new bone formation (24). These findings suggest that it may be necessary to block IL-23 in the pre-clinical phase, before the disease phenotype becomes clinically apparent. Based on these findings in the rats, it could be hypothesized that there may be a pre-clinical disease stage also in human SpA where specific immune pathways are already activated before the disease becomes clinically manifest (**Figure 1**). This concept of a pre-clinical “at-risk phase” phase is already well-established in a different form of chronic arthritis, rheumatoid arthritis (RA) (29–31). Better understanding of the molecular alterations present in the at-risk phase of RA have resulted in initial treatment trials aiming to prevent onset of disease (32, 33).

For long, it has been speculated that extra-musculoskeletal tissue inflammation in SpA contributes to the initiation of arthritis and spondylitis and subsequent development of full-blown SpA. To address the potential tissue-specific role of IL-23/IL-17 in both joint and extra-musculoskeletal tissues, it would be ideal to examine these target tissues, however, this is challenging due to the relative lack of tissue samples that can easily be obtained from affected lesions. This is even more challenging in individuals who are at increased risk of SpA, before the onset of clinically manifest disease. Animal data could provide a good basis for future human studies allowing better understanding of the disease development, and on how extra-musculoskeletal tissues are involved in initiation of this disease. Increased understanding of the molecular pathways active in this very early stage of disease in SpA might identify novel potential treatment targets or even provide a basis for preventive treatment strategies aiming to further improve outcome for SpA patients.

Here, we provide an overview of findings from various experimental SpA animal models indicating how the IL-23/IL-17 axis is implicated in the initiation and progression of disease, with a focus on the major tissues involved in SpA pathogenesis.

## Experimental Models of SpA

Evidence from studies in animal models, human expression studies, and SpA genetic association studies, has indicated that the IL-23/IL-17-axis contributes to pathogenesis of chronic arthritis, spondylitis, and associated inflammatory manifestations (34).

**Figure 2:**
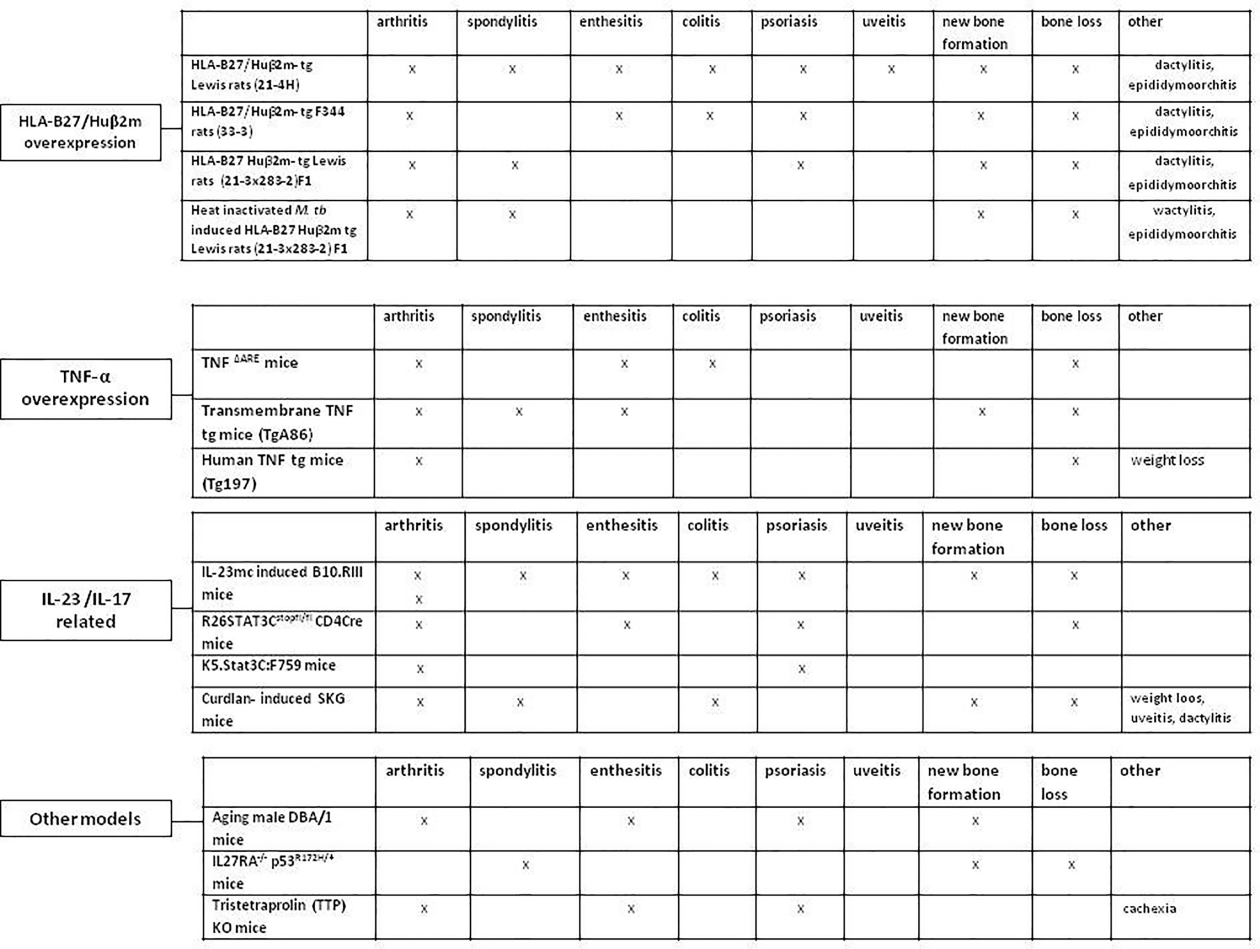
Animal models resembling human spondyloarthritis. HLA-B27/Huβ2m- tg, HLA-B27/human β2 microglobulin transgenic rat; TNF-α, Tumor necrosis factor alpha; IL-23, Interleukin-23; IL-17, Interleukin-17; M. tub, Mycobacterium tuberculosis; IL-23mc, Interleukin-23 minicircle; Human TNF tg mice, Human tumor necrosis factor transgenic mice.

There are several animal models that bear resemblance to human SpA (**Figure 2**) (35–37). These models differ in genetic modifications, disease mechanism and pathologic features. Collectively, they contribute to dissecting the pathogenic processes in SpA disease development and progression. Herein, we first briefly summarize the most commonly used experimental models of SpA. An thorough overview of these SpA animal models has been previously presented by Viera de Sousa et al. (35). There are more recent animal models developed since, which we are highlighting here as well (37).

### HLA-B27/Huβ2m Overexpression

HLA-B27 is the major genetic risk factor for SpA (38, 39), and overexpression of HLA-B27 resulted in clear SpA-like features in rats (38), but not in mice (40). Over time, the HLA-B27 transgenic (tg) rat model has progressed from the HLA-B27/human β2 microglobulin (hβ2m) (tg) Lewis (21-4H) rats, characterized by orchitis, colitis and hind limbs arthritis, with psoriasis in up to 50% of the rats, to the HLA-B27/hβ2m-tg F344 (33-3) rats with similar clinical manifestations but with earlier disease onset. This was followed by the HLA-B27/hβ2m-tg Lewis rats (21-3 x 283-2)F1 line with lower HLA-B27 copy numbers, in which all male rats develop orchitis, followed by the development of arthritis (4–6 months age) in 70% of all male rats, and spondylitis (7–9 months age) in 50% of them (41). These rats also show signs of peripheral and axial new bone formation. Most recently, immunizing the (21-3 x 283-2)F1 rats with heat-inactivated *Mycobacterium tuberculosis* (*M.tb*) has been demonstrated to synchronize and accelerate the disease onset in both male and female rats. Arthritis and spondylitis development is visible in 80-100% of the rats 2-3 weeks post *M.tb* immunization (35).

### TNF-α Overexpression

TNF-α is one of the key cytokines in SpA pathogenesis and there are several animal models that are based on TNF-α overexpression. In the TNF^ΔARE^ mice, the overexpression of murine TNF-α leads to chronic peripheral polyarticular synovitis, enthesitis and colitis, without psoriasis, osteoproliferation or spinal involvement (42–44). The colitis severity varies from asymptomatic histological inflammation to severe ileitis depending on housing conditions. In contrast, mice that overexpress transmembrane TNF (tmTNF) (TgA86) (45) do develop new bone formation together with signs of peripheral arthritis and spondylitis, without gut or skin involvement (35). The human TNF-(Tg197) tg mice is a model characterized by destructive polyarticular synovitis (including sacroiliitis), with no spinal involvement, new bone formation, gut or skin inflammation (46). The differences in clinical presentation between the soluble and tmTNF overexpression models indicate that tmTNF drives the key clinical phenotype and pathologic bone formation of SpA.

## IL-23/IL-17 Related Animal Models of SpA

Animal models played an important role in implicating the IL-23/IL-17 axis in SpA pathogenesis. In the IL-23 minicircle (mc) induced B10.RIII mice model, high systemic IL-23 expression results in axial and peripheral enthesitis with entheseal new bone formation, with no destructive synovitis at day 35 (47). Another model that is considered IL-23/IL-17 dependent is the SKG model with the ZAP-70^W163C^ mutation. This ZAP-70^W163C^ mutation is downstream of the T cell receptor signaling complex. This mutation affects positive and negative selection of T cells. Moreover, it alters the generation and function of CD25+CD4+ natural regulatory T (Treg) cells (48). SKG mice develop arthritis, enthesitis, spondylitis, peripheral new bone formation and Crohn’s disease-like ileitis, but without clear signs of axial new bone formation. The disease onset can be synchronized by one IP injection of the fungal wall component curdlan. In this model, curdlan activates IL-23 release, inducing mucosal dysregulation and IL-17 and IL-22 cytokine expression, driving the SpA phenotype (49, 50).

Recently, IL-27RA^-/-^ p53^R172H/+^ mice have been demonstrated to show SpA-like disease (37). As a result of knocking out IL-27RA, these mice lack the inhibitory effect of IL-27 on Th17 lineage differentiation. IL-27 is a member of the IL-12 cytokine family and is known to down regulate IL-23, RAR-related orphan receptor (ROR)-γt, and Th17 differentiation (12, 47, 51), while p53 is known to negatively regulate osteoblast differentiation, bone formation and remodeling pathway (52). The IL-27RA^-/-^p53^R172H/+^ mice demonstrate minimal axial inflammation and bone loss, with pathological bone formation at the intervertebral discs (37). Moreover, these mice also show skin inflammation (neutrophilic dermatitis) and additional organ pathology including chronic kidney nephropathy (37, 53).

Other models reflecting the importance of IL-17A production in SpA disease pathogenesis are the STAT3 overexpression models. STAT3 is a key regulatory factor inducing differentiation of naïve CD4^+^ T cells into Th17 cells (54). The group of Yang Lu introduced the R26STAT3C^stopfl/fl^ CD4Cre mouse model reflecting SpA with signs of psoriasis, driven by a specific hyperactive T cell-STAT3C allele (55, 56). The disease manifestations include spontaneous development of psoriatic skin lesions, enthesitis/tendinitis, and arthritis (57). Peri-articular bone erosion and osteopenia were also observed, without signs of new bone formation.

Similarly, overexpression of STAT3 in the K5.STAT3C:F759 murine model also results in spontaneous severe psoriatic cutaneous lesions and peripheral erosive arthritis (36).

## Other Models

The aging male DBA/1 mice spontaneously develop brief arthritis, dactylitis, and enthesitis. In 10-12 weeks old mice progressive endochondral bone formation has been shown but still without axial or extra-articular pathologic changes (58, 59).

More recently, the Tristetraprolin (TTP) KO model has been shown to develop a severe, systemic inflammatory syndrome, with destructive arthritis, conjunctivitis, dermatitis, osteopenia, myeloid hyperplasia and cachexia (60). TTP is a RNA binding protein that has important endogenous anti-inflammatory effects, through destabilizing mRNAs encoding pro-inflammatory cytokines and suppressing their biosynthesis (e.g. TNF-α). The clinical and immunological phenotype associated with TTP deficiency was dependent on the IL-23/IL-17A axis (61). Most of the expressed phenotype was prevented using anti- (TNF-α) antibody treatment (60).

## Involvement of IL-23/IL-17 axis in the Initiation Phase of Disease

Although the major clinical presentation of SpA involves the joints (axial or peripheral), innate immune activation by either microbial stress caused by barrier dysfunction at the gut or the skin, or by mechanical stress caused by microtrauma at the entheses, is thought to trigger disease onset (27). The exact underlying mechanisms are not yet fully understood (**Figure 2**). To gain more insight into the disease initiation phase, of SpA, when first pathological molecular alterations occur, we assessed the result of IL-23 and IL-17 dysregulation in SpA experimental models. We highlight studies in which the disease initiation in SpA may be explained by IL-23/IL-17 activation systemically or locally in the entheseal areas close to the joints. Moreover, we will discuss other potential initiation sites of SpA by summarizing how gut and skin inflammation may relate to development of joint inflammation.

Sherlock et al. have shown that induction of high systemic IL-23 levels can induce enthesitis before development of new bone formation and synovial joint destruction. After induction of disease with IL-23mc, mice develop paw swelling at day 5-10 with entheseal inflammation (47). Although F4/80+ macrophages and myeloperoxidase (MPO)**+** neutrophils were observed in the entheseal inflammatory infiltrate, IL-23 responsive RORγt+CD3+CD4-CD8- entheseal resident T cells, later identified as γδ T cells (62) were shown to be important for initiating inflammation, as this specific subset of T cells were shown to produce IL-17A and IL-17F, and IL-22 (47) and inhibition of IL-17 and/or IL-22 did decrease disease severity. Depletion of Th17 cells was shown not to change the disease course, indicating that indeed the resident entheseal T cells play an important role in driving the disease onset. In this model, the entheseal inflammation was followed by expansion of periosteal osteoblasts by day 18, accompanied by a molecular new bone formation gene expression profile. Entheseal new bone formation was observed at day 35 (47).

In the SKG model, curdlan (consisting of the fungal wall component beta-glucan) signaling *via* the dectin 1 pattern-recognition receptor, that specifically recognizes beta-glucans, was applied as a trigger of a SpA phenotype (49, 63). Curdlan injection induces ileal IL-23 secretion, which in turn provokes a state of mucosal dysregulation and cytokine imbalance. The mice develop (histological) inflammation at the axial and peripheral entheses 1 week after curdlan induction followed by development of clinical enthesitis, sacroiliitis, peripheral arthritis and colitis and uveitis with increased serum levels of IFN-γ, TNF-α, IL-6 and IL-17A. The surge of IFN-γ, IL-6 and IL-17A cytokines was shown to be IL-23 dependent (50, 64). The disease manifestations were shown to be IL-23 dependent and partially IL-17 dependent (64). Early IL-23 inhibition using anti-IL-23 mAb before disease induction in SKG mice resulted in a clear clinical improvement in these mice. There were no histological signs of arthritis and spondylitis (50, 64).

The importance of IL-23 in disease initiation was also found in the accelerated heat-inactivated *M.tb* induced HLA-B27 tg rat model. It was shown that there is a short increase in *IL-23R* expression and *RORC* gene expression shortly after immunization (24). Blocking IL-23R after immunization but before onset of clinical manifestations completely prevented arthritis and spondylitis development. This prophylactic treatment significantly suppressed the lymph nodes and splenic mRNA expression of various downstream cytokines, e.g. *Il-17A, Il-22, Mmp*3 and *Ccl20*. However, expression of other pro-inflammatory cytokines such as *Il-17F, Ifn-γ, Tnf-α*, and *Il-6* were not affected by IL-23R blocking. Although IL-23 was clearly implicated in disease initiation, the source and the cell type(s) responsive to IL-23 in this model need to be elucidated (24).

In the same accelerated HLA-B27tg rat model, prophylactic anti-IL-17A treatment significantly delayed the development and decreased the severity of spondylitis and arthritis. On histology there was less inflammation and less destruction in axial and peripheral joints in anti–IL-17A treated rats, but the disease onset was not prevented (65). This is in contrast to the findings with anti-IL-23R blocking therapy, as anti-IL23R did prevent the disease onset.

Strikingly, in the B10.RII mice, the use of IL-17Amc DNA over-expression, as well as IL-17A blockade revealed no major role for IL-17A in driving arthritis in the B10.RIII model (47). Consistent with that, IL-17A deficiency in the IL-17A^-/-^ SKG mice only resulted in a moderate reduction of the SKG phenotype (64). Altogether, the data from these SpA models indicate that IL-17A is likely not the only cytokine that contributes to the initiation of IL-23 dependent arthritis.

In addition to IL-17A, IL-22, as a downstream cytokine of IL-23, was shown to be relevant for the severity of enthesitis in both the IL-23mc and SKG mice model (47, 64, 66). *Ex vivo* gene expression profiling revealed the induction of murine *Il-6, Il-22* and *Cxcl1* by day 5 of IL-23 expression in the IL23mc model (47). Blocking IL-22 in SKG mice for 8 weeks from the first moment of clinical manifestations reduced Achilles tendon enthesitis, similar to the reduced severity of enthesitis observed in the IL-17A^-/-^ SKG mice (64).

The aforementioned animal studies link IL-23 responsive cells to initiation of disease and development of clinical SpA manifestations. In humans innate immune cells have been reported to express IL-23R with inducible IL-17A/F expression (67). Recently also presence of conventional CD4+ and CD8+ T cells have been reported in human entheses, which upon stimulation could produce TNF-α and IL-17A. However, IL-17 production here is independent of IL-23 (67–69). If these cells could play a role in the onset of SpA in humans needs further investigation. This link between IL-23 responsive cells and initiation of disease in animal models direct further in depth characterization of both innate and adaptive immune cells from the moment of triggering of inflammation (possibly in the pre-clinical disease stage) to onset of clinically manifest spondyloarthritis to further elucidate which IL-23 responsive cells drive pro-inflammatory cytokine production, including IL-17A, and which cells drive IL-17 production independently of IL-23.

Altogether, IL-23 plays an important role in inducing SpA pathology as revealed in the different models. If immune dysregulation of IL-23 is also present in the pre-clincal phase of human SpA needs further investigation. If in the future we can identify individuals at very high risk of developing SpA, who would qualify for preventive treatment strategies, then anti-IL23 treatment could be suggested to be the first candidate to be tested as a preventive medicine. However, currently we lack good predictive tools that could identify those individuals at increased risk of developing SpA who would qualify for such a treatment strategy.

## The Gut-Joint Axis in the Initiation of Spondyloarthritis

SpA patients often present with gut inflammation which led to the hypothesis that inflammation at the gut mucosa may initiate SpA pathogenesis (70, 71). Theoretically, gut inflammation could result in loss of intestinal barrier integrity allowing microbes and dietary antigens to enter into the bloodstream, and trigger immune dysregulation leading to joint inflammation (72–74). An in-depth review discussing the gut-joint axis in SpA was recently provided by Gracey et al. (71). Here we will discuss current literature on the potential crucial role of the IL-23/IL-17 axis in the transition of inflammation from the gut to the joint.

IL-17 has been considered to play a dual role in gut homeostasis. Whereas IL-23-independent IL-17A production by innate(-like) immune cells is considered to be protective in colitis by maintaining barrier function in the intestines (75, 76), IL-23-dependent IL-17A production by Th17 cells results in gut inflammation (76, 77). This might explain the opposing effects observed in clinical trial in active crohn’s disease where IL-17A inhibition resulted in worsening of colitis, but treatment with anti-IL-12/IL-23p40 as well as p19 inhibition (78) improved inflammation (79). In addition to IL-17A, IL-17F has been demonstrated to promote inflammation in the intestines through its effect on the intestinal microbiome in mice (80). A study in experimental colitis provided evidence that inhibition of both IL-17A and IL-17F was necessary to attenuate colitis (81). IL-22 is another key player in intestinal host defense and maintaining mucosal homeostasis, which adds further complexity to role of the IL-17/IL-23 axis in gut inflammation. It induces direct intestinal expression of complement C3 and mucin genes, shares in the clearance of pathogens (82–84), *via* facilitating the production of cytokines and chemokines that mediate innate cell recruitment to infection sites (84). Furthermore, IL-22 has been shown to regulate hemopexin production, in order to impair bacterial growth (85).

Gut inflammation is a prominent feature in the high copy numbers HLA-B27 tg rat models (21-4H and 33-3 line) (38). Colitis is seen at 6 weeks age and increased *Il-23p19* expression occurred at the start of colitis associated with increased *Il-17A*, next to *Il-1, Il-6*, and *Tnf-α* expression. IL-23p19 and IL-17A transcripts were localized to CD11c+ antigen presenting cells and CD4+ T cells, respectively (86). This was accompanied by increased HLA-B27 expression and signs of HLA-B27 misfolding with an unfolded protein response (86). Mechanistically, it is thought that the increased HLA-B27 expression in activated macrophages results in an unfolded protein response with ER stress promoting IL-23 expression (86–88). This IL-23 could then induce pathogenic IL-17A production by Th17 cells. Ciccia et al. showed human evidence for HLA-B27’s role in gut inflammation. They have observed that HLA-B27 misfolding occurs in the gut of AS patients and is associated with activation of autophagy. Autophagy appears to induce intestinal expression of IL-23 in the human gut (89).

Furthermore, Utriainen et al. (90) reported a significantly decreased subset of intestinal dendritic cells (DCs), which are involved in maintaining self-tolerance, in the mesenteric lymph nodes (MLNs) and gut draining lymph of HLA-B27-tg (33-3) rats. The deficiency of these tolerogenic DCs in combination with the improved ability of bone marrow-derived DCs to stimulate IL-17 production by CD4+ T cells could induce an immune mediated inflammatory response involving the IL-23/IL-17 axis. Possibly, the dysbalance in proinflammatory and toloregenic DCs as well as the HLA-B27 misfolding as observed in these experimental animal models (86, 91, 92), might also be relevant for induction of disease in HLA-B27+ individuals.

In HLA-B27 tg (33-3) rats it was observed that HLA-B27 homodimer expression on various lymphocyte populations increases between week 6-23 of age, and is accompanied by colitis development and expansion of IL17+ CD4+ T cells and TNF+ CD4+ T cells. Of interest, presence of HLA-B27 homodimer expression on monocytes in gut draining MLN, but not on splenocytes, co-occurred with the expansion of Th17 cells, when colitis was first observed. Besides HLA-B27 misfolding, HLA-B27 homodimerization is proposed as another mechanism by which HLA-B27 can contribute to IL-17 production, which has been reviewed previously (93–95). HLA-B27 is normally present on the cellular surface with β_2_m as a heterodimer, however when presented as a homodimer, this homodimer can interact with killer-cell immunoglobulin-like receptors (KIRs) on the surface of NK cells and T cells. This could result in direct activation of NK cells and T cells and aberrant cytokine production, contributing to an enhanced activation of the IL-23/IL-17 pathway (96).

The evidence for the role of the IL-23/IL-17 axis in colitis preceding joint inflammation was further provided by Glatigny (91). In the HLA-B27 tg (33-3 line) rats Glatigny et al. observed increased IL-17+TNF+ T cells in MLN which coincided with colitis, and later this was followed by the occurrence of the same cell types in PLN at the same time as the onset of arthritis. The increase in IL-17A+TNF+ T cells was paralleled by elevated mRNA expression levels of several genes indicating a Th17 phenotype (i.e. *Il-21, Il-22*, and *Rorc*) and increased serum levels of IL-17A (91).

These studies add to the concept that immune dysregulation in the gut precedes the onset of other clinical manifestations of SpA, possibly mediated by dysbiosis and/or colitis. They suggest that IL-23 produced in the gut could influence local activation of lymphoid cells in the entheses (47, 64). Various innate immune lymphoid cells able to produce these cytokines have been implicated in human SpA pathogenesis (reviewed in Gracey et al.) *(* 71). If the IL-17+ T cells present in the gut mucosa are indeed pathogenic and if they play a role in the migration of immune cells from the gut to the joints needs further investigation. That lymphocytes can possibly egress from gut to joint prior to disease onset was suggested in a study in TNF^ΔARE^ mice, reported as a conference abstract, where lymphocytes were shown to traffic from the colon to the joints (97). Ciccia et al. provided data, supporting active gut-joint trafficking in human axSpA. They showed an expanded gut-derived IL-17+ and IL-22+ ILC3 population expressing α4β7 in the peripheral blood, synovial fluid and inflamed bone marrow of patients (98).

Dysbiosis is an alteration in the composition of complex commensal or microbiota that might be induced by a wide range of environmental factors (99). Related to colitis, dysbiosis is suggested as an important factor that drives pathologic immune pathways. In HLA-B27 tg rats (33-3) it was shown that gut metabolomic changes are already present before the onset of colitis suggesting that these alterations might be an initiating event, however if these changes affect the IL-23/IL-17 pathway was not investigated (100). Gill et al. investigated microbial dysbiosis in different HLA-B27 rat strains and found no common overlapping microbial pattern but showed that the genetic background determined the dysbiotic microbial pattern present in each model. Nevertheless, microbial dysbiosis (regardless of the pattern) provoked similar immune response with an obvious increase in IL-23 and IL-17, as well as IL-1, IFN-γ, and TNF-α cytokines coinciding with colitis as early as 2 months of age in the various strains (101).

Gut microbes triggering the immune system were also observed in the SKG mouse models of SpA. Observations in the ZAP-70^W163C^ mutant SKG mice and TLR-4-deficient SKG mice have confirmed the role of gut microbiota and therelated colitis in SpA. In contrast to the germ free SKG mice, that are free of all microorganisms including those that are typically found in the gut, the curdlan treated specific pathogen fee (SPF) SKG mice show increased constitutive *Il-23* expression specifically in ileal tissue, associated with ER stress marker expression and MLNs cytokine production including *Il-6, Tnf-α, Ifn-γ* and *Il-17A (* 64). Involvement of gut microbes was reported by Rehaume et al. who showed that (intraperitoneal) curdlan administration induced acute systemic inflammation with IL-6, TNF, MCP-1 production and neutrophil recruitment disregarding the presence of the SKG allele or microbiota, with the response diminishing earlier in the germ-free animals. Neutrophil IL-17A production in the peritoneal cavity was shown to be microbiota dependent, as was ER-stress induced increased ileal IL-23 expression, MLN IL-17A expression, goblet cell loss, and ileitis development. In contrast, the development of associated arthritis and spondylitis was not crucially dependent on the microbiota profile, but incidence of arthritis and its severity clearly differed with various microbial profiles (102). These studies provide a link of microbial innate immune triggering in gut to development of SpA features. These microbiome alterations may be further induced by the tissue inflammation in the gut, making it difficult to determine what the initiating factor was: the dysbiosis or the tissue inflammation? (103). In human SpA, dysbiosis has been seen both in patients (104) as well as in healthy HLA-B27+ individuals (105), and it remains speculative if dysbiosis lowers the threshold for gut inflammation, and the onset of SpA.

## Skin

Psoriasis is an extra-musculoskeletal manifestation often seen in SpA patients (4), in particular in a subtype of SpA, psoriatic arthritis (PsA). The proinflammatory role of IL-17A in the development of articular and cutaneous manifestations of PsA has been consistently established in animal models as well as in human PsA. Here, we highlight the animal models that describe the concomitant presence of psoriasis and musculoskeletal manifestations.

In wild type C57BL/6J mice, as early as 1 day after IL-17A mc gene transfer, clinical psoriatic changes were observed, accompanied by an inflammatory infiltrate of neutrophils. Moreover, gene expression of murine keratin 16 (*K16*), a marker of keratinocyte hyper-proliferation was highly expressed. It remains to be assessed which cells are responsible for both the skin inflammation as well as the joint and bone pathology (106).

Recently, additional novel animal models demonstrating the cutaneous, articular, and associated bone changes of psoriatic arthritis were developed. Yang et al. showed in the R26STAT3C^stopfl/fl^ CD4Cre mice SpA like disease, which is driven by an amplified Th17 response downstream of T cell-specific expression of a hyperactive STAT3C allele (55, 56). Animals developed psoriasis-like skin lesions after 5 weeks with increased infiltration of single IL-17+, IL-22+ or combined IL-17/IL-22+ CD4+ T cells. The associated Achilles tendon enthesitis showed increased cell infiltration with both IL-23R and RORγt double positive CD4+ T cells. These findings imply that Th17 cells play an important role in the development of skin and entheseal inflammation (56).

The importance of STAT3 in psoriatic skin inflammation was also shown when specifically activated in keratinocytes in the K5.STAT3C:F759 mice model. Yamamoto et al. demonstrated that early occurrence of arthritis in K5.STAT3C:F759 mice was due to coincident skin inflammation induced by a hyper STAT3 activation. Mice showed increased serum IL-6 and IL-17A levels. IL-17A expression was elevated compared with F759 mice in inflamed joints, which contain increased numbers of CCR6+ CD4+ T cells compared to the LN compartment (36).

Although these 3 models clearly show involvement of the IL-23/IL-17 axis in development of psoriasis and arthritis, development of arthritis was not preceded by psoriasis but occurred simultaneously.

Skin inflammation preceding joint inflammation was addressed in F759 (not harboring the K5.STAT3C transgene) mice. These mice are known to spontaneously develop arthritis after 12-18 month. It was also shown that forced induction of psoriasis-like lesions by imiquimod application in these mice significantly accelerated arthritis development, suggesting that psoriatic inflammation facilitated arthritis development, possibly by activating the IL-17 pathway, as IL-17+ γδ T cells as well as Th17 cells were present in the inflamed skin. However, other options should not be ruled out as imiquimod can also have important systemic effects (36). Recently a study reported (currently only available in abstract) that psoriasis-like skin inflammation in C57/Bl6 male mice induced mild synovial joint inflammation. Similar results were obtained after dextran sodium sulphate (DSS) colitis induction in these mice (107).

These preliminary data indicate that the skin is a candidate location for triggering joint pathology. Besides follow-up studies in animal models resembling PsA, future longitudinal studies in patients with psoriasis at increased risk of development of PsA, are expected to provide further insight into the mechanisms triggering joint disease in PsA. Future studies could benefit from applying state of the art molecular imaging in combination with (serial) tissue biopsy analysis to repetitively assess tissue inflammation.

## Conclusion and Future Perspectives

Literature on the animal models resembling human SpA indicates an overlapping and distinctive role of IL-17A and IL-23 in the initiation of disease. There is initial evidence for inflammation at gut and skin to precede joint inflammation in animal models of SpA with involvement of the IL-23/IL-17 axis. Importantly, in accordance with the insights from the *M*.*tb* HLA-B27 study (24, 27), IL-23 plays an important role in the onset of SpA-like pathology in many animal models, even before clinical joint manifestations are present, which is in clear contrast with the absence of any significant clinical effect as observed when blocking IL23R in diseased rats (24) or with blocking of IL-23p19 in AS patients (25). The data in animal models may help us to understand which pathways are dysregulated early in the disease process, in the pre-clinical phase, and if these dysregulations differ from observations in established disease.

While animal models provide an ideal basis for in-depth molecular analysis of target tissues in SpA to elucidate causal relationships between the upregulation of specific immune pathways and development of key SpA features, complimentary studies performing serial advanced molecular imaging or bio-sample collection in individuals with an increased risk of developing SpA or PsA, or in patients who have been recently diagnosed withSpA, are essentially required if we want to translate findings in animal models of SpA to human disease. The goal of studying the preclinical stage of the disease would be to find better diagnostic markers to allow an earlier diagnosis and open up opportunities for preventive treatment strategies (108–110). If IL-23 dysregulation in the pre-clinical phase of disease, as observed in various animal models, can be confirmed in human pre-clinical SpA, it could be speculated that IL-23 targeting may be effective in preventing disease, in contrast to the lack of effect observed with IL-23 blocking in established SpA. There is more recent evidence that Th17 cells in the synovial tissue are polyfunctional and produce multiple proinflammatory cytokines that synergize with IL-17A. Treatments targeting Th17 cells, or affecting multiple cytokines that synergize with IL-17A in pathogenic immune responses could also be considered for preventive trials, Whether these pathways are upregulated early in the disease pathogenesis remains to be elucidated. As an example, potential novel treatments such as TYK2 inhibition (108), mTOR inhibition by rapamycin (110), and PI3Kδ inhibition (109) have been shown *in vitro* and/or *in vivo* animal models to affect multiple cytokines relevant for SpA pathogenesis. Lastly, it remains to be studied if preclinical treatment in patients could prevent or affect pathologic bone formation, as this remains an unmet treatment need in SpA patients.

The IL-23/IL-17 axis is clearly implicated in disease initiation in SpA. However, which specific innate (-like) or adaptive immune cells are responsible for the pathogenic IL-17A production in the various target tissues, is still incompletely understood. Moreover, the effects of the plasticity of the Th17/Treg cells in initiating inflammation as well as the pathogenic role of pathways that enable IL-23 independent IL-17A production are still incompletely understood and prompt further research both in human SpA as in animal models resembling SpA.

To conclude, animal models have improved our understanding of the IL-23/IL-17 axis in SpA and continue to allow us to gain novel insights on SpA pathogenesis. Greater understanding of disease initiation in SpA animal models could support human preclinical SpA studies, This may provide a basis for better future preventive approaches for our patients.

## Author Contributions

MM, SC, and MS: substantial contributions to conception and design, MM, SC and MS: drafted the article, over viewed it critically for important intellectual content and given final approval of the final version. All authors contributed to the article and approved the submitted version.

## Funding

MM is supported by a Dutch Arthritis Society grant (number 19-1-206).

## Conflict of Interest

MS: Consultancy fees/speaker fees from Abbvie, Novartis and Eli Lilly, MSD. Research support from Novartis, Eli Lilly, UCB, Janssen.

The remaining authors declare that the research was conducted in the absence of any commercial or financial relationships that could be construed as a potential conflict of interest.

## Abbreviations

ADAM17, A disintegrin and metalloprotease 17; AIA, adjuvant-induced arthritis; AxSpA, Axial spondyloarthritis; CIA, collagen-induced arthritis; DCs, Dendritic cells; HLA-B27/Huβ2m- tg, HLA-B27/human β2 microglobulin transgenic rat; IL-23R, Interleukin 23 receptor; IL-23, Interleukin 23; IL-17A, Interleukin 17A; ILC, Innate lymphoid cells; LN, Lymph node; MLN, Mesenteric lymph node; NF-κB, Nuclear factor kappa-Beta; PsA, Psoriatic arthritis; NSAIDs, Non-steroidal anti-inflammatory drugs; PLN, Popliteal lymph node; PRR, Pattern recognition receptors; PsO, Psoriasis; TACE, TNF-α converting enzyme; TLR, Toll like receptors; TNF-α, Tumour necrosis factor alpha; TNFR1, Tumor necrosis factor receptor 1; TTP, Tristetraprolin; RANKL, Receptor activator of nuclear factor κB ligand; STAT3, Signal transducer and activator of transcription 3; SpA, Spondyloarthritidis; SPF, Specific pathogen free; UPR, Unfolded protein response.
